# LGBTQ+ Inclusive Policies, Nurse Job Outcomes, and Quality of Care in Hospitals

**DOI:** 10.1001/jamanetworkopen.2025.1765

**Published:** 2025-03-25

**Authors:** Hyunmin Yu, Matthew D. McHugh, José A. Bauermeister, Tari Hanneman, Karen B. Lasater

**Affiliations:** 1 Center for Health Outcomes and Policy Research, School of Nursing, University of Pennsylvania, Philadelphia; 2 Human Rights Campaign Foundation, Washington, DC

## Abstract

**Importance:**

Despite emphasis on the establishment of inclusive hospital policies, the impact of these policies on employees and organizations remains unknown.

**Objective:**

To evaluate the association between inclusive policies for lesbian, gay, bisexual, transgender, queer or questioning, and other sexual and gender minority (LGBTQ+) and nurse job outcomes as well as nurse-reported quality of care.

**Design, Setting, and Participants:**

This cross-sectional study analyzed 4 survey datasets from 2021: the RN4CAST-NY/IL, including registered nurses from New York and Illinois, and the Healthcare Equality Index (HEI) data. The HEI evaluates and scores US health care facilities that voluntarily participate based on their LGBTQ+ inclusivity in policies, such as nondiscrimination policies and LGBTQ+ inclusive clinical services. The study used American Hospital Association Annual Survey data for hospital characteristics and Magnet organization data to classify hospitals by Magnet status. Data analyses were performed in December 2024.

**Main Outcomes and Measures:**

Nurse job outcomes included burnout and job dissatisfaction. Quality of care outcomes included nurses’ perceptions of care quality and their likelihood of recommending their hospital. The independent variable was LGBTQ+ Healthcare Equality Leader (HEI Leader) status, which signified hospitals with the highest levels of LGBTQ+ inclusion. Multilevel logistic regression models included nurse-level (age, race and ethnicity, gender, and years of experience at the current hospital) and hospital-level (Magnet status, size, teaching status, specialized service capability, and ownership) covariates.

**Results:**

A total of 7343 nurses (mean [SD] age, 44.9 [13.4] years; 6584 [89.6%] women) from 111 hospitals were included in the study. Nurses in hospitals with HEI Leader status had lower odds of high burnout (adjusted odds ratio [AOR], 0.69; 95% CI, 0.52-0.92) and lower odds of job dissatisfaction (AOR, 0.62; 95% CI, 0.45-0.86) compared with those in hospitals without the status. They also had higher odds of reporting excellent or good quality of care (AOR, 1.83; 95% CI, 1.23-2.73) and higher odds of recommending their hospital (AOR, 1.72; 95% CI, 1.19-2.50).

**Conclusions and Relevance:**

In this cross-sectional study, nurses in hospitals with high LGBTQ+ inclusion reported more favorable job outcomes and care quality. Hospitals should understand that implementing LGBTQ+ inclusive policies goes beyond compliance or diversity; it is essential for improving the work climate, enhancing staff well-being, and optimizing care delivery.

## Key Points

**Question:**

Are LGBTQ+ inclusive hospital policies associated with nurse job outcomes and quality of care?

**Findings:**

In this cross-sectional study of 7343 nurses from 111 hospitals, nurses in hospitals with high LGBTQ+ inclusion reported lower burnout, reduced job dissatisfaction, better care quality, and a greater willingness to recommend their hospitals.

**Meaning:**

Hospitals should recognize that LGBTQ+ inclusive policies are not only about compliance or diversity but also crucial for improving work climate, staff well-being, and care delivery.

## Teaser

This cross-sectional study evaluates the association between lesbian, gay, bisexual, transgender, queer or questioning, and other sexual and gender minority (LGBTQ+) inclusive policies in hospitals and nurse job outcomes and quality of care.

## Introduction

The implementation of inclusive policies for lesbian, gay, bisexual, transgender, queer or questioning, and other sexual and gender minority (LGBTQ+) inddividuals is gaining prominence given the persistent discrimination faced by LGBTQ+ patients and employees in health care settings.^1,2,3,4^ Since 2007, the Healthcare Equality Index (HEI) has been used to assess various aspects of these policies in health care organizations, including nondiscrimination policies, inclusive clinical services, and employee benefits tailored to LGBTQ+ needs.^5^ Studies on the HEI have primarily focused on its association with patient satisfaction^6,7^ and hospital characteristics, including a hospital’s Magnet status.^8,9^ Although the HEI has been instrumental in guiding health care organizations toward LGBTQ+ inclusivity, empirical evidence is needed to evaluate whether and to what extent these inclusive policies are associated with better outcomes for health care employees and patients.

Nurse job outcomes, including burnout and job dissatisfaction, driven by system-level factors, such as poor work environments,^10,11^ are closely linked to quality of care^12,13,14^ and patient outcomes.^14,15^ Studies have reported that various hospital characteristics—including a hospital’s Magnet status,^16^ size,^17^ and ownership^18^—have been associated with nurse job outcomes and nurse-reported quality of care measures. However, organizational policies related to inclusion have not been investigated, even though these policies can play a crucial role in creating equitable and supportive environments, as emphasized by inclusive organization theories.^19,20,21^

Theories on inclusive organization suggest that an inclusive climate—characterized by policies supporting diversity and inclusion—can significantly impact both employee well-being and organizational outcomes.^19,20,21^ These models propose that organizations that foster inclusivity enhance employee satisfaction, engagement, and retention while also improving organizational performance, including innovation and productivity.^19,20,21^ In health care, an inclusive climate fostered by LGBTQ+ inclusive policies may reduce stressors related to discrimination and foster a sense of belonging among staff, leading to lower burnout and job dissatisfaction among nurses.^19,20,21^ Additionally, when nurses feel supported and valued, they are more likely to perceive the care they provide as high quality and to recommend their hospital to others.^22,23^

**Figure 1.**
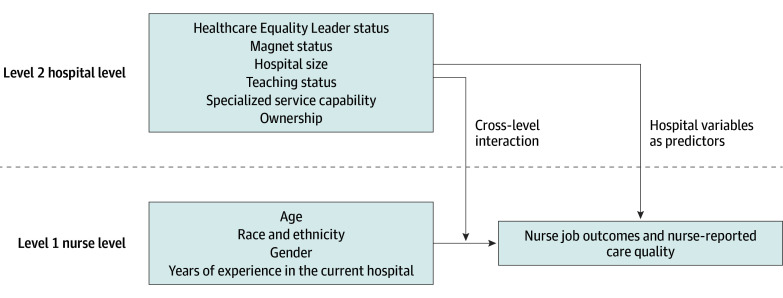
The Hypothesized Model of Multilevel Approach We hypothesized that a hospital’s LGBTQ+ Healthcare Equality Leader (HEI Leader) status would be positively associated with nurse job outcomes and nurse-reported care quality, accounting for nurse-level (age, gender, race and ethnicity, and years of experience at the current hospital) and hospital-level (Magnet status, teaching status, hospital size, specialized service capability, and ownership) variables. Additionally, we hypothesized an interaction between HEI Leader status and nurse demographics, suggesting that the effect of HEI Leader status on job and care quality outcomes may vary by nurse demographics.

This study evaluates the association between LGBTQ+ inclusive policies and nurse job outcomes as well as nurse-reported quality of care. Additionally, it examines whether this association varies based on nurses’ demographics (Figure 1). Given the American Hospital Association’s (AHA’s) emphasis on the importance of inclusive hospital policies,^24^ understanding these dynamics and their association with tangible employee and organizational outcomes is crucial for guiding policy and practice in hospital settings.

## Methods

### Study Design

This cross-sectional study is a secondary analysis of 4 datasets: the RN4CAST-NY/IL, the HEI, the AHA Annual Survey, and Magnet organizations. The RN4CAST-NY/IL study collected data between April and June 2021 by surveying more than half a million actively licensed registered nurses in New York and Illinois via emails obtained from state board nursing licensure lists.^13^ The survey aimed to assess nurse demographics and job outcomes,^13^ with nurses serving as informants on hospital characteristics to ensure strong representation of hospitals and their environments.^25^ Given our study’s focus on hospital settings, we included 14 896 nurses who reported working in hospitals and provided specific hospital names from a total of 70 072 respondents.

**Figure 2.**
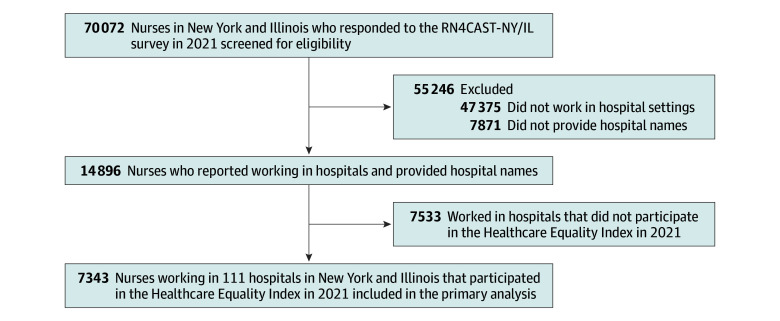
Sampling Diagram The figure illustrates the process of selecting a sample of nurses working in hospitals in New York and Illinois that participated in the Healthcare Equality Index in 2021.

We used the 2022 HEI data collected in 2021^26^ and matched them with hospitals included in the RN4CAST-NY/IL survey. A total of 112 distinct hospitals in New York and Illinois participated in the HEI in 2021. Our analysis included 7343 nurses from 111 of these hospitals, as nurses in 1 hospital did not participate in the RN4CAST-NY/IL survey (Figure 2). On average, study hospitals had 66 nurse respondents to the RN4CAST-NY/IL survey. The 2021 AHA Annual Survey data were used to account for hospital characteristics,^27^ and the Magnet organization data were used to categorize study hospitals by Magnet status as of 2021.^28^ The study protocols were approved by the University of Pennsylvania institutional review board, and all nurses provided written informed consent before their participation. The Strengthening the Reporting of Observational Studies in Epidemiology (STROBE) reporting guideline was followed.

### Measures

#### Job Outcomes 1: High Burnout

Burnout was assessed using the 9-item emotional exhaustion subscale from the Maslach Burnout Inventory.^29,30^ Participants rated each item on a 7-point Likert scale, with responses ranging from never (score of 0) to every day (score of 6). Sum scores were used, with possible values ranging from 0 to 54. In our analytic sample, the Cronbach α = 0.95. A score of 27 or higher indicated high burnout.^31^ With this cutoff, the variable was treated as binary, with 0 indicating no high burnout and 1 indicating high burnout.

#### Job Outcomes 2: Job Dissatisfaction

Job dissatisfaction was assessed using a single-item measure.^13^ Participants rated their level of satisfaction with their job by responding to the question, “Overall, how satisfied are you with your job?” on a 4-point Likert scale, with 1 indicating very satisfied; 2, moderately satisfied; 3, moderately dissatisfied; and 4, very dissatisfied. The variable was treated as binary, with 0 indicating very satisfied and moderately satisfied and 1 indicating moderately dissatisfied and very dissatisfied.

#### Quality-of-Care Outcomes 1: Nurse-Reported Quality of Care

Nurse-reported quality of care was assessed through a single item that has been highly associated with objective measures of patient outcomes, including mortality.^32,33^ The question, “How do you rate the quality of nursing care in your workplace?” was answered on a 4-point Likert scale, with 1 indicating excellent; 2, good; 3, fair; and 4, poor. This variable was treated as binary, with 0 indicating fair and poor and 1 indicating excellent and good.

#### Quality-of-Care Outcomes 2: Nurses’ Recommendation of Their Hospital to Family or Friends

Nurses’ recommendation of their hospital to family or friends was assessed with a single item: “Would you recommend where you work to your family and friends if they needed health care?” Responses were rated on a 4-point Likert scale, with 1 indicating definitely yes; 2, probably yes; 3, probably not; and 4, definitely not.^12^ This variable was treated as binary, with 0 indicating definitely not, probably not, and probably yes and 1 indicating definitely yes. This binary categorization was used to distinguish strong endorsement (definitely yes) from less definitive responses.

#### Independent Variable: Hospitals’ HEI Leader Status

The independent variable was a hospital’s LGBTQ+ Healthcare Equality Leader (HEI Leader) status. The HEI is a survey designed to evaluate the LGBTQ+ inclusivity of policies and practices in US health care facilities that voluntarily participate by completing an extensive online survey and providing supporting documentation from hospital administrators. The Human Rights Campaign Foundation validates their responses and assigns scores from 0 to 100 based on detailed criteria, with 0 indicating the lowest level of overall inclusion for LGBTQ+ patients, visitors, and employees and 100 indicating the highest level of overall inclusion for them.^34^ The HEI score is composed of 4 domains. The first, nondiscrimination and staff training, evaluates policies on patient and employee nondiscrimination and staff training in LGBTQ+ care, scoring from 0 to 40, with 0 indicating no nondiscrimination policies and training on LGBTQ+ inclusive care and 40 indicating explicit policies and comprehensive staff training. The second, patient services and support, assesses the availability of clinical services for LGBTQ+ individuals, including transgender-specific services, scoring from 0 to 30, with 0 indicating no LGBTQ+ inclusive clinical services or inclusive sex and gender data collection and 30 indicating comprehensive services and data collection. The third, employee benefits and policies, evaluates benefits for LGBTQ+ employees, such as coverage for LGBTQ+ specific care, scoring from 0 to 20, with 0 indicating no equal benefits for same-sex partners, no transgender care coverage, and no support for LGBTQ+ employees and 20 indicating fully equitable benefits, transgender care coverage, and robust support systems. The fourth, patient and community engagement, measures outreach efforts to the LGBTQ+ community, scoring from 0 to 10, with 0 indicating no support for local LGBTQ+ events and noninclusive patient surveys and 10 indicating active support for events and fully inclusive patient surveys addressing LGBTQ+ needs. In our study, the Cronbach α = 0.85. Points are deducted for discriminatory policies or anti-LGBTQ+ incidents. Facilities scoring 100 points, indicating the highest level of LGBTQ+ inclusion, were designated as HEI Leaders. Hospitals in the study were categorized as 0 for no HEI Leader status and 1 for HEI Leader status.

#### Level 1 Covariates (Nurse Level)

We included sociodemographic factors previously associated with burnout and job dissatisfaction among nurses. These factors include race and ethnicity,^35,36^ age,^37,38^ gender,^39^ and years of experience at the current hospital.^40^ Age and years of experience at the current hospital were treated as continuous variables. Nurses self-reported their race and ethnicity on the survey as part of the nurse demographics. The race question included the following options: American Indian or Alaska Native, Asian, Black or African American, Native Hawaiian or Other Pacific Islander, and White. The ethnicity question offered 2 options: Hispanic or non-Hispanic. On the basis of the minority categorization from the National Institute on Minority Health and Health Disparities,^41^ the authors created a binary variable for race and ethnicity by combining 2 questions, with 0 representing non-Hispanic White and 1 representing racial and ethnic minority groups, including those who identified as any Hispanic ethnicity and American Indian or Alaska Native, Asian, Black or African American, or Native Hawaiian or Other Pacific Islander races. The gender question offered options for man, woman, and other, with a specification field. After reviewing written responses, we added a category for transgender and gender-diverse individuals alongside the cisgender categories.

#### Level 2 Covariates (Hospital Level)

We included structural hospital characteristics previously associated with nurse job outcomes and HEI performance, including a hospital’s Magnet status,^8,16^ hospital size,^6,17^ specialized service capability,^42,43^ and teaching status.^6^ We also included ownership as an operational hospital characteristic because it shapes operational priorities and resources, influencing care and inclusivity. Magnet status was binary, with 0 indicating non-Magnet and 1 indicating Magnet as of 2021. Teaching status was assessed using the ratio of resident physicians and fellows to hospital beds, categorizing hospitals as nonteaching, minor teaching (≤1:4), or major teaching (>1:4).^44^ Specialized service capability was classified as high for hospitals offering open-heart surgery or major transplants and low for those lacking these services.^45^ Hospital size was categorized as small (≤100 beds), medium (101-250 beds), or large (>250 beds).^43^ Because no for-profit hospitals were in our study, ownership was classified as federal or nonprofit, including nonfederal government and nongovernmental entities.

### Statistical Analysis

We used Stata software, version 17 (StataCorp LLC) to analyze the associations between HEI Leader designation, nurse job outcomes, and nurse-reported care quality measures. Given the hierarchical structure of the data, with nurses nested within hospitals, and the binary nature of the outcomes, we used a 2-level logistic regression model (level 1 indicating nurse level and level 2 indicating hospital level) to investigate the association of nurse- and hospital-level factors with nurse job outcomes and care quality.^46,47^

We initiated our analysis by specifying the null model, which included only the dependent variable. Next, we specified the level 1 model, which included the dependent variable and level 1 variables. Then, we specified the level 2 model, incorporating the dependent, level 1, and level 2 variables. Before model specification, we calculated generalized variance inflation factors for each independent variable to assess multicollinearity. All variables had low to moderate values, allowing inclusion of both level 1 and level 2 variables in the level 2 model. Finally, we conducted exploratory analyses of cross-level interactions between nurse demographics and hospitals’ HEI Leader status.

Among the 7343 nurses, 5415 had complete responses for all study variables, whereas 1928 nurses had at least 1 missing value (missingness rate, 26.3%). The highest number of missing responses (n = 1532) was related to nurses’ recommendations of their hospital. Although the exact mechanism for the missing data was unclear, our analysis indicated that the missingness was unrelated to the observed data. Given the high rate of missing data, we used multiple imputations using chained equations.^48^ Before conducting multiple imputation, we performed the Little test of missing completely at random to evaluate whether the assumptions required for multiple imputation were met in our dataset. The test produced a *P* = .19, supporting the use of multiple imputation. Sensitivity analyses comparing multiple imputation results with those from complete case analyses showed no significant differences. In our study, a 2-sided *P* < .05 was considered statistically significant.

## Results

Among the 111 hospitals, 84 (75.7%) were in New York and 27 (24.3%) were in Illinois (Table 1). The mean (SD) total HEI score was 95.6 (11.9), ranging from 20 to 100. A total of 85 hospitals (76.6%) held HEI Leader status, and 48 (43.2%) held Magnet status. Additionally, 87 (78.3%) were teaching hospitals, 70 (63.1%) had more than 250 beds, 42 (37.8%) had high specialized service capability, and 95 (85.6%) were nonprofit hospitals.

**Table 1.** Hospital and Nurse Characteristics

Characteristic	No. (%)
**Hospital characteristics (n = 111)**	
State	
New York	84 (75.7)
Illinois	27 (24.3)
Total Healthcare Equality Index score, mean (SD)^a^	95.6 (11.9)
LGBTQ+ Healthcare Equality Leader status	
Yes	85 (76.6)
No	26 (23.4)
Magnet status	
Yes	48 (43.2)
No	63 (56.8)
Teaching status	
Major	49 (44.1)
Minor	38 (34.2)
None	24 (21.6)
Hospital size	
Large (>250 beds)	70 (63.1)
Medium (101-250 beds)	33 (29.7)
Small (≤100 beds)	8 (7.2)
Specialized service capability	
High	42 (37.8)
Low	69 (62.2)
Ownership	
Nonprofit	95 (85.6)
Federal	16 (14.4)
**Nurse characteristics (n = 7343)**	
Age, mean (SD), y	44.9 (13.4)
Race and ethnicity	
Non-Hispanic White	4524 (61.6)
Racial and ethnic minority^b^	2819 (38.4)
Time in current hospital, mean (SD), y	11.5 (11.2)
Gender	
Women	6584 (89.6)
Men	754 (10.3)
Transgender or gender diverse	5 (0.1)
High burnout status^c^	
Yes	3180 (43.3)
No	4163 (56.7)
Job dissatisfaction	
Very or moderately dissatisfied	1710 (23.3)
Very or moderately satisfied	5633 (76.7)
Quality of care	
Poor or fair	1336 (18.2)
Good or excellent	6007 (81.8)
Likelihood to recommend their hospital	
Definitely yes	2991 (40.7)
Definitely not, probably not, or probably yes	4352 (59.3)

Abbreviation: LGBTQ+, lesbian, gay, bisexual, transgender, queer or questioning, and other sexual and gender minority.

^a^ Score ranges from 0 to 100; 0 indicates the lowest level of overall inclusion for LGBTQ+ patients, visitors, and employees, and 100 indicates the highest level of overall inclusion.

^b^ Includes those who identified as any Hispanic ethnicity or American Indian or Alaska Native, Asian, Black or African American, or Native Hawaiian or Other Pacific Islander races.

^c^ Score of 27 or higher on the emotional exhaustion subscale of the Maslach Burnout Inventory.

Among the 7343 nurses, the mean (SD) age was 44.9 (13.4) years; 6584 (89.6%) identified as women, 754 (10.3%) as men, and 5 (0.1%) as transgender or gender diverse. A total of 4524 (61.6%) identified as non-Hispanic White, and 2819 (38.4%) were from racial and ethnic minority backgrounds (including those who identified as any Hispanic ethnicity or American Indian or Alaska Native, Asian, Black or African American, or Native Hawaiian or Other Pacific Islander races). They had a mean (SD) of 11.5 (11.2) years of experience at their current hospital (Table 1). A total of 3180 (43.4%) reported high burnout, and 1710 (23.3%) were moderately or very dissatisfied with their jobs. Additionally, 1336 (18.2%) rated the quality of care as poor or fair, whereas 2991 (40.7%) indicated they would definitely recommend their hospital to family and friends.

### Multilevel Analyses

Nurses in hospitals with HEI Leader status had lower odds of experiencing high burnout compared with those without the status (adjusted odds ratio [AOR], 0.69; 95% CI, 0.52-0.92; *P* = .01) (Table 2). Nurses in Magnet hospitals had lower odds of experiencing high burnout compared with those in non-Magnet hospitals (AOR, 0.67; 95% CI, 0.57-0.80; *P* < .001). Nurses in hospitals with high specialized service capability had higher odds of experiencing high burnout compared with those in hospitals with low capability (AOR, 1.28; 95% CI, 1.05-1.56; *P* = .01). Increasing age was associated with lower odds of experiencing high burnout, with each additional year of age reducing the odds by 3% (AOR, 0.97; 95% CI, 0.96-0.97; *P* < .001). The same series of multilevel models were applied to job dissatisfaction, quality of care, and nurse recommendations of their hospital, all demonstrating significant associations with a hospital’s HEI Leader status. Nurses in hospitals with HEI Leader status had lower odds of experiencing job dissatisfaction compared with those without the status (AOR, 0.62; 95% CI, 0.45-0.86; *P* = .005) (Table 2). Additionally, nurses in hospitals with HEI Leader status had higher odds of reporting the quality of care as excellent or good (AOR, 1.83; 95% CI, 1.23-2.73; *P* = .003) and higher odds of definitely recommending their hospital to family and friends (AOR, 1.72; 95% CI, 1.19-2.50; *P* = .004) compared with those in hospitals without HEI Leader status (Table 3). No cross-level interaction between HEI Leader status and nurse demographics for these outcomes was statistically significant.

**Table 2.** Multilevel Logistic Model With Nurse Job Outcomes as the Dependent Variable

Variable	High burnout		Job dissatisfaction	
	Adjusted odds ratio (95% CI)	*P* value	Adjusted odds ratio (95% CI)	*P* value
Intercept	4.60 (3.23-6.54)	<.001	1.17 (0.78-1.76)	.44
Fixed effects: nurse-level variables				
Age	0.97 (0.96-0.97)	<.001	0.97 (0.97-0.98)	<.001
Years of experience in the current hospital	1.00 (0.99-1.01)	.11	1.00 (1.00-1.01)	.24
Race and ethnicity				
Non-Hispanic White	1 [Reference]	NA	1 [Reference]	NA
Racial and ethnic minority^a^	1.00 (0.90-1.11)	.98	0.99 (0.87-1.12)	.82
Gender				
Women	1 [Reference]	NA	1 [Reference]	NA
Men	0.97 (0.83-1.13)	.68	1.14 (0.95-1.36)	.16
Transgender and gender diverse	0.58 (0.09-3.58)	.55	0.67 (0.07-6.18)	.32
Fixed effects: hospital-level variables				
LGBTQ+ Healthcare Equality Leader status				
Yes	0.69 (0.52-0.92)	.01	0.62 (0.45-0.86)	.005
No	1 [Reference]	NA	1 [Reference]	NA
Magnet status				
Yes	0.67 (0.57-0.80)	<.001	0.66 (0.54-0.80)	<.001
No	1 [Reference]	NA	1 [Reference]	NA
Hospital size				
Large (>250 beds)	1 [Reference]	NA	1 [Reference]	NA
Medium (101-250 beds)	1.02 (0.81-1.29)	.86	1.07 (0.82-1.39)	.63
Small (≤100 beds)	0.97 (0.76-1.24)	.80	1.06 (0.60-1.87)	.84
Teaching status				
None	1 [Reference]	NA	1 [Reference]	NA
Minor	1.51 (0.95-2.40)	.08	1.09 (0.81-1.46)	.57
Major	0.99 (0.79-1.24)	.95	1.07 (0.81-1.41)	.64
Specialized service capability				
High	1.28 (1.05-1.56)	.01	1.54 (1.22-1.94)	<.001
Low	1 [Reference]	NA	1 [Reference]	NA
Ownership				
Nonprofit	1 [Reference]	NA	1 [Reference]	NA
Federal	0.70 (0.50-1.05)	.09	0.64 (0.40-1.03)	.07
Random effects				
Intercept (hospital identification)	0.09 (0.05-0.15)	<.001	0.12 (0.07-0.21)	<.001

Abbreviations: LGBTQ+, lesbian, gay, bisexual, transgender, queer or questioning, and other sexual and gender minority; NA, not applicable.

^a^ Racial and ethnic minority includes those who identified as any Hispanic ethnicity and American Indian or Alaska Native, Asian, Black or African American, or Native Hawaiian or Other Pacific Islander races.

**Table 3.** Multilevel Logistic Model With Quality of Care Measures as the Dependent Variable

Variable	Quality of care		Recommendation of their hospital	
	Adjusted odds ratio (95% CI)	*P* value	Adjusted odds ratio (95% CI)	*P* value
Intercept	1.00 (0.62-1.64)	.99	0.08 (0.05-0.12)	<.001
Fixed effects: nurse-level variables				
Age	1.01 (1.00-1.02)	<.001	1.02 (1.02-1.03)	<.001
Time in the current hospital	1.00 (1.00-1.02)	.07	1.01 (1.00-1.01)	.003
Race and ethnicity				
Non-Hispanic White	1 [Reference]	NA	1 [Reference]	NA
Racial and ethnic minority^a^	0.82 (0.71-0.94)	.006	0.95 (0.84-1.06)	.35
Gender				
Women	1 [Reference]	NA	1 [Reference]	NA
Men	0.80 (0.66-0.97)	.03	0.88 (0.74-1.04)	.14
Transgender and gender diverse	1.07 (0.10-11.04)	.95	1.06 (0.15-7.36)	.96
Fixed effects: hospital-level variables				
LGBTQ+ Healthcare Equality Leader status				
Yes	1.83 (1.23-2.73)	.003	1.72 (1.19-2.50)	.004
No	1 [Reference]	NA	1 [Reference]	NA
Magnet status				
Yes	3.01 (2.33-3.89)	<.001	3.04 (2.41-3.83)	<.001
No	1 [Reference]	NA	1 [Reference]	NA
Hospital size				
Large (>250 beds)	1 [Reference]	NA	1 [Reference]	NA
Medium (101-250 beds)	1.16 (0.85-1.59)	.36	1.08 (0.82-1.41)	.59
Small (≤100 beds)	0.72 (0.39-1.35)	.31	0.79 (0.46-1.37)	.41
Teaching status				
None	1 [Reference]	NA	1 [Reference]	NA
Minor	1.03 (0.72-1.47)	.88	1.13 (0.83-1.55)	.43
Major	0.99 (0.71-1.39)	.96	1.06 (0.79-1.43)	.68
Specialized service capability				
High	0.74 (0.56-0.97)	.03	0.75 (0.59-0.95)	.02
Low				
Ownership				
Nonprofit	1 [Reference]	NA	1 [Reference]	NA
Federal	2.31 (1.30-4.09)	.004	1.68 (0.99-2.82)	.05
Random effects				
Intercept (hospital identification)	0.24 (0.15-0.38)	<.001	0.22 (0.14-0.32)	<.001

Abbreviations: LGBTQ+, lesbian, gay, bisexual, transgender, queer or questioning, and other sexual and gender minority; NA, not applicable.

^a^ Racial and ethnic minority includes those who identified as any Hispanic ethnicity and American Indian or Alaska Native, Asian, Black or African American, or Native Hawaiian or Other Pacific Islander races.

## Discussion

Our findings highlight the significant association of LGBTQ+ inclusive policies with nurse job outcomes and care quality. Nurses in hospitals with HEI Leader status, recognized for their commitment to the highest levels of LGBTQ+ inclusion, reported lower burnout, reduced job dissatisfaction, better quality of care, and greater willingness to recommend their hospitals to family and friends. Hospitals should understand that implementing LGBTQ+ inclusive policies is not only about compliance or diversity; it is crucial for improving the overall work climate. This, in turn, enhances staff well-being and care delivery. LGBTQ+ inclusive policies should not be viewed as a nice-to-have feature or a token gesture. Instead, it is a vital component in shaping the overall organizational culture and is significantly associated with both employee satisfaction and the quality of care provided.

Our results align with theories on inclusive organizations,^19,20,21^ demonstrating that working in hospitals with LGBTQ+ inclusive policies is positively associated with nurse job outcomes and care quality. These outcomes not only reflect nurse well-being but also suggest broader organizational benefits, including improved patient outcomes. A healthy and satisfied nursing workforce is vital for delivering high-quality care, directly impacting patient safety, satisfaction, and overall health care outcomes.^49,50^ This emphasizes the importance of continued efforts to foster inclusivity within hospitals as a strategy for achieving both workforce well-being and patient care excellence.

Although our study demonstrates the positive outcomes associated with LGBTQ+ inclusive policies, we found no significant cross-level interaction between HEI Leader status and nurse demographics. This finding may suggest that the benefits of inclusive policies are broadly experienced across different demographic groups of nurses, without varying effects based on characteristics such as gender, age, or race and ethnicity. However, this finding warrants further exploration, as there may be variability in other states with different legal or political environments or among different subpopulations. Future studies should include broader geographic locations and more diverse nurse populations, with attention to specific racial and ethnic, sexual orientation, and socioeconomic subgroups. Expanding sample diversity will allow researchers to better investigate whether certain groups experience differential benefits from LGBTQ+ inclusive policies.

Structural hospital characteristics were significantly associated with nurse job outcomes and nurse-reported care quality in our study. Consistent with prior studies,^16,51^ Magnet hospitals showed more favorable nurse outcomes than non-Magnet hospitals, likely due to their commitment to nursing excellence and supportive work environments.^52^ A recent study^8^ found that Magnet hospitals outperformed non-Magnet hospitals in the HEI. Future research should explore whether hospitals recognized for both nursing excellence and LGBTQ+ inclusion have a synergistic association on employee and patient outcomes. In contrast, hospitals with high specialized service capability were negatively associated with nurse job outcomes, suggesting that advanced care settings may increase nurse stress due to higher patient acuity, care complexity, and workloads.

### Strengths and Limitations

Given the limited research on the association of inclusive hospital policies with job outcomes and care quality, despite the emphasis placed on such policies by the AHA,^24^ this study’s strength lies in its theory-based exploration of these associations. However, the study has several limitations. First, its cross-sectional design limits causal inferences. Although associations between HEI Leader status and job and care quality outcomes are supported by a theoretical framework, causality cannot be established. Second, the voluntary nature of HEI participation introduces selection bias, as only approximately 13% of more than 6000 US hospitals participated in 2021. This may skew results, as participating hospitals are likely to already have supportive policies. Future studies should investigate how participation in the HEI itself impacts employee and patient outcomes compared with nonparticipation, given the voluntary nature of the program. Additionally, the study relies on data from only 2 US states, both of which have relatively progressive LGBTQ+ laws and policies compared with other states.^53^ This geographic limitation may affect the generalizability of the findings to other regions with different political and legal environments. Third, the surveys were conducted during the COVID-19 pandemic, which significantly stressed health care systems and workers. Future studies should reassess these outcomes in a postpandemic context.

## Conclusions

In this cross-sectional study, nurses in hospitals with high LGBTQ+ inclusion reported lower burnout, reduced job dissatisfaction, better care quality, and a greater willingness to recommend their hospitals. Hospitals should understand that implementing LGBTQ+ inclusive policies goes beyond compliance or diversity; it is essential for improving the work climate, enhancing staff well-being, and optimizing care delivery.

## Notes

Supplement.
Data Sharing Statement
