# Supplementary material for: LGBTQ+ Inclusive Policies, Nurse Job Outcomes, and Quality of Care in Hospitals
Source: JAMA Netw Open. 2025 Mar 25;8(3):e251765. doi: 10.1001/jamanetworkopen.2025.1765 (PMC11937948; doi:10.1001/jamanetworkopen.2025.1765)
Supplement: Supplement. — Data Sharing Statement [file jamanetwopen-e251765-s001.pdf]

Data Sharing Statement

Yu. LGBTQ+ Inclusive Policies, Nurse Job Outcomes, and Quality of Care in Hospitals. *JAMA Netw Open*. Published March 25, 2025. doi:10.1001/jamanetworkopen.2025.1765

Data
Data available: No
